# Direct arterial injection of hyperpolarized ^13^C‐labeled substrates into rat tumors for rapid MR detection of metabolism with minimal substrate dilution

**DOI:** 10.1002/mrm.26628

**Published:** 2017-02-12

**Authors:** Steven Reynolds, Stephen Metcalf, Edward J. Cochrane, Rebecca C. Collins, Simon Jones, Martyn N.J. Paley, Gillian M. Tozer

**Affiliations:** ^1^ Academic Unit of Radiology, Department of Infection, Immunity and Cardiovascular Disease University of Sheffield Sheffield United Kingdom; ^2^ Tumour Microcirculation Group, Department of Oncology and Metabolism University of Sheffield Sheffield United Kingdom; ^3^ Department of Chemistry, Dainton Building University of Sheffield Brook Hill Sheffield United Kingdom; ^4^ Present address: King's College London, British Heart Foundation Centre of Excellence, Cardiovascular Division London United Kingdom

**Keywords:** intra‐arterial, P22 tumor, hyperpolarization, ^13^C‐pyruvate, ^13^C‐glucose, combretastatin

## Abstract

**Purpose:**

A rat model was developed to enable direct administration of hyperpolarized ^13^C‐labeled molecules into a tumor‐supplying artery for magnetic resonance spectroscopy (MRS) studies of tumor metabolism.

**Methods:**

Rat P22 sarcomas were implanted into the right inguinal fat pad of BDIX rats such that the developing tumors received their principle blood supply directly from the right superior epigastric artery. Hyperpolarized ^13^C‐molecules were either infused directly to the tumor through the epigastric artery or systemically through the contralateral femoral vein. Spectroscopic data were obtained on a 7 Tesla preclinical scanner.

**Results:**

Intra‐arterial infusion of hyperpolarized ^13^C‐pyruvate increased the pyruvate tumor signal by a factor of 4.6, compared with intravenous infusion, despite an approximately 7 times smaller total dose to the rat. Hyperpolarized glucose signal was detected at near‐physiological systemic blood concentration. Pyruvate to lactate but not glucose to lactate metabolism was detected in the tumor. Hyperpolarized ^13^C‐labeled combretastatin A1 diphosphate, a tumor vascular disrupting agent, showed an in vivo signal in the tumor.

**Conclusions:**

The model maximizes tumor substrate/drug delivery and minimizes T_1_ relaxation signal losses in addition to systemic toxicity. Therefore, it permits metabolic studies of hyperpolarized substrates with relatively short T_1_ and opens up the possibility for preclinical studies of hyperpolarized drug molecules. Magn Reson Med 78:2116–2126, 2017. © 2017 The Authors Magnetic Resonance in Medicine published by Wiley Periodicals, Inc. on behalf of International Society for Magnetic Resonance in Medicine. This is an open access article under the terms of the Creative Commons Attribution License, which permits use, distribution and reproduction in any medium, provided the original work is properly cited.

## INTRODUCTION

Hyperpolarized dissolution dynamic nuclear polarization (dDNP) is an established technology for in vivo monitoring of metabolism by magnetic resonance spectroscopy (MRS) and MRI, without the interfering background signals experienced in ^1^H MRS 1, 2. After dissolution, the hyperpolarized substrate is rapidly administered to experimental animals, and signals are acquired from metabolites within a few multiples of the T_1_ relaxation time, before the signal decay becomes too significant. The most commonly used hyperpolarized substrate, ^13^C_1_‐pyruvate, has a reported T_1_ of approximately 50 s for in vitro aqueous solutions, whereas in vivo values, including those for extracted blood, have been estimated in the range of 17 to 30 s 3, 4, 5. The search for other metabolic targets for hyperpolarization has focused on molecular groups with inherently long T_1_, such as those with carbonyl groups and deuterated compounds. Unfortunately, many metabolically active molecules and drug candidates have short T_1_s of 1 to 20 s, which are difficult to apply in preclinical discovery research because of the strong correlation between the observed T_1_ and signal enhancement 6. An alternative is the development of molecular probes in which the spin system is capable of being put into a singlet state 7, which considerably lengthens the magnetization relaxation time. However, this approach has so far established very few suitable probes.

Typically, hyperpolarized substrates are administered systemically to mice or rats through an intravenous injection. However, a potential pitfall with dDNP hyperpolarization is the supra‐physiological quantities of substrate required to acquire an acceptable signal‐to‐noise ratio (SNR) in the tissue of interest. For the most common hyperpolarized molecules, the dose is well‐tolerated (eg, pyruvate and urea), or their injected concentration is close to the endogenous blood concentration (eg, lactate 8 and glucose 9). No adverse reactions have been reported for these substrates. However, use of such high concentrations of substrates/metabolites perturbs tissue biochemistry and, if hyperpolarized drug candidates are to be used, then the required concentration for successful MR signal acquisition is likely to be well above their maximum tolerated doses.

This problem would be substantially reduced if the hyperpolarized substrate could be administered directly to the tissue of interest, thus avoiding substrate dilution and minimizing the time required to reach the tissue. Indeed, this has been done in ex vivo perfusions of rat hearts for metabolism studies 10, 11. For cancer studies, specialized tumor models have been developed in which most of the tumor blood supply arises from a single supplying artery and draining vein 12, 13. Such “tissue‐isolated” models have been used recently for studying the circadian effects on tumor metabolism 14 and tumor response to vascular targeting agents 15. However, no work has been reported on their use in MRS/MRI studies of tumor metabolism using hyperpolarized substrates. Here, we report the signal enhancement achievable for ^13^C_1_‐pyruvate and ^13^C_u_‐glucose‐d7 using direct injection into a tumor‐supplying artery. Additionally, in vivo monitoring of drug metabolism was evaluated for the first time by administering a custom synthesized hyperpolarized ^13^C‐labeled version of the vascular disrupting pro‐drug, combretastatin A‐1‐diphosphate (CA1‐P) 16.

## METHODS

### Animal Preparation

Animal experiments were conducted in accordance with the UK Animals Act 1986, with local ethical approval and following published ethical guidelines 17. The surgical procedure for growing tissue‐isolated tumors in the rat inguinal fat‐pad was slightly modified from our previously described method 12. Briefly, 9 to 13‐week‐old in‐bred immuno‐competent BDIX rats from an in‐house specific pathogen free colony (mostly male; see fig. legends for details) were anesthetized using isoflurane (5% induction; 2% maintenance; balance O_2_). The right inguinal fat pad was exposed and a portion bluntly dissected from the skin to allow its surgical isolation together with the proximal portion of the superior epigastric artery and vein. A total of 1 × 10^6^ dissociated early passage syngeneic P22 fibrosarcoma cells 18 in 50 μL Hanks balanced salt solution, obtained from a donor animal, were injected into the isolated portion of the fat pad using a 25‐gauge needle. The needle track was temporarily clamped shut to seal the fat and prevent escape of the cells. The isolated fat pad containing the tumor cells was loosely sutured to the bulk of the remaining fat pad to allow movement of the growing tumor, while preventing twisting of the pedicle containing the superior epigastric artery and vein. The incision was closed with three horizontal mattress sutures (5/0 vicryl). Two‐percent lidocaine HCl (Hamelin Pharmaceuticals, Gloucester, UK) was used for immediate topical pain relief, and buprenorphine (Vetergesic, Alstoe Veterinary, York, UK; 0.03 mg/kg/day for 2 days postoperatively) for extended analgesia. Rats were kept on a 12 h light/dark cycle, with temperature maintained at 19 to 23°C. They were housed singly after surgery and provided with moistened and normal rat chow and water ad libitum.

**Figure 1:**
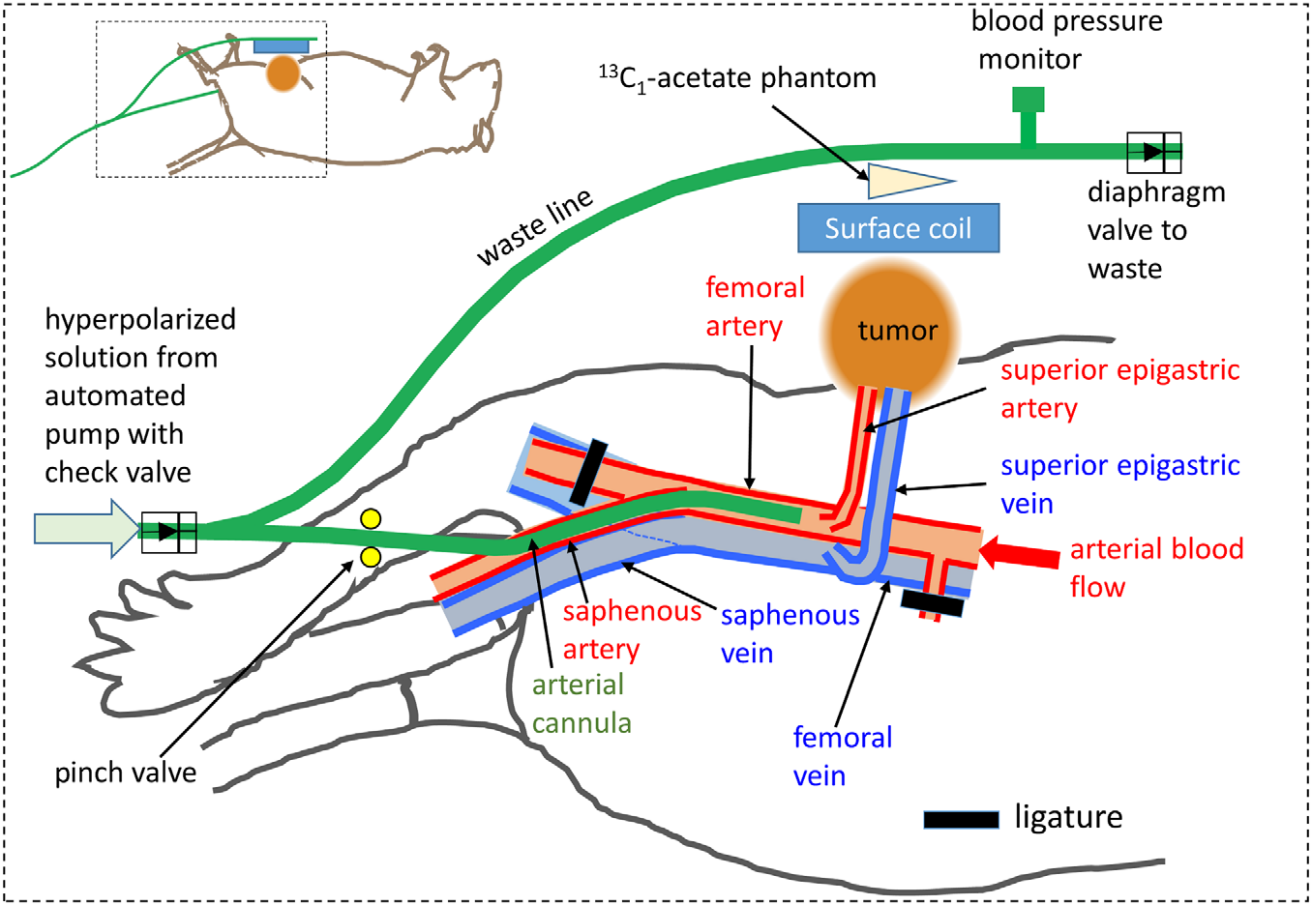
Representation of the tumor implant, approximating its location in the inguinal cleft of a BDIX rat and showing the vascular morphology and arrangements of the arterial cannula, waste line, surface coil, phantom, blood pressure monitor, and diaphragm valve (not to scale). The arterial cannula is positioned with its tip just distal to the branch of the superior epigastric artery from the femoral artery. The distal portion of the femoral artery and side branches are ligated such that infusate from the arterial cannula is washed into the tumor‐supplying superior epigastric artery via normal arterial blood flow. The waste line passes above the ^13^C_1_‐acetate phantom and surface coil, with a branch for blood pressure monitoring. Direction of flow of the hyperpolarized solution is controlled via the pinch and diaphragm valves. Inset shows the approximate position of the tumor, cannula, waste line, and surface coil with respect to the whole rat.

The developing tumor receives its principle blood supply directly from the right superior epigastric artery branching from the right femoral artery, with the superior epigastric vein draining the tumor (Fig. 1). Tumors were allowed to grow for approximately 7 days before MR scanning or optical imaging. For preparatory surgery prior to imaging, anesthesia was maintained using 1 to 2% isoflurane delivered at 2 L/min in 1:1 oxygen:nitrous oxide. For MR scanning and optical imaging, the same concentration of isofluorane was used in 22 to 24% O_2;_ balance N_2_. Surgery involved right saphenous artery cannulation up to the superior epigastric artery branch of the right femoral artery for substrate delivery (Figs. 1 and 3). Throughout surgery and subsequent procedures, the animal's temperature was maintained at 37°C using a homeothermic blanket system (Harvard apparatus, Cambridge, UK), with supplementary heated air for MR scanning. Respiration rate was measured using a surface transducer (SA Instruments Inc, Stony Brook, NY, USA), and mean arterial blood pressure was measured with a pressure transducer (CWE Inc, Ardmore, PA, USA) in line with the arterial cannula (Fig. 1), to enable monitoring of animal welfare while in the magnet. To prevent the wound from drying out during scanning, a saline drip line was directed onto the wound site. The saline was heated by placing the drip line under the heating blanket just before entering the wound site, and was supplied at 1 mL/h using a syringe pump. The wound was covered with sterile gauze and protected with Parafilm. Animals were sacrificed after scanning.

### Optical Imaging

Macroscopic and microscopic optical imaging was used for preliminary studies to visualize the arterial delivery of compounds to tumor tissue. Following tumor growth and the surgical procedure described previously, either 5 mg/mL methylene blue (methylthioninium chloride; Proveblue, Marseille, France) or 0.24 mL undiluted fluorescent 15 µm diameter polystyrene microbeads (Fluospheres; excitation 450 nm; emission 480 nm; Fisher Scientific, Loughborough, UK) were infused via the saphenous artery cannula, at 1 L/min using an infusion pump. For methylene blue infusion, the preparation was viewed under a Nikon SMZ 1000 (Melville, NY, USA) operating microscope and the infusion recorded at 13 frames/s for approximately 15 s following the start of infusion (Jenoptik ProgRes XTcore5 digital camera, Indigo Scientific, Baldock, UK). For fluorescent microbeads, tumors were excised immediately after infusion, embedded in optimum cutting temperature compound and snap‐frozen, using isopentane cooled on dry ice, and stored at −80°C. Ten‐micrometer‐thick cryo‐sections were stained by immuno‐fluorescence for a rat endothelial cell surface antigen (RECA‐1; Serotec, Kidlington, UK) with Alexa Fluor 555 tagged anti‐mouse secondary (ThermoFisher Scientific, Loughborough, UK). Cell nuclei were stained using DAPI in the mounting medium (Vector Laboratories, Peterborough, UK). Tumor sections were imaged using either an Olympus BX61 (Tokyo, Japan) fluorescence microscope for single fields or a Leica AF6000 (Wetzlar, Germany) inverted fluorescence microscope for composite images of whole‐tumor sections.

### Hyperpolarization

For the hyperpolarization of pyruvate and glucose, OXO63 trityl radical (Oxford Instruments, Abingdon, UK) and DOTAREM (Guerbet, Roissy, France) were added to either neat ^13^C_1_‐pyruvic acid (Sigma Aldrich, UK) or 3.3 to 3.45 M ^13^C_u_‐glucose‐d7 (Sigma Aldrich, UK) in D_2_O, to final concentrations of 15 mM (OXO63 trityl radical) and 1.5 mM (DOTAREM) for both pyruvate and glucose.

A total of 35.5 ± 0.5 mg (∼27 μL) of the prepared pyruvic acid sample was inserted into a HyperSense dDNP system (Oxford Instruments) and polarized up to more than 90% of maximum polarization (40–60 min). The hyperpolarized sample was dissolved with superheated 40 mM HEPES buffer solution and transferred to an automated injection system 19 in 4.5 s, for infusion into the rat. For the pyruvate dissolution a predetermined aliquot of 2.0 M NaOH solution was added to the injection system to neutralize the pyruvic acid acidity. The final injectate concentration of pyruvate was approximately 80 mM. Glucose was polarized to approximately 90% of maximum polarization and transferred to the injection system, as for pyruvate, with the exception of using deuterated water in the HEPES buffer solution. Quantities of polarized glucose were varied for the desired final injectate concentration (10–156 mM).

**Figure 2:**
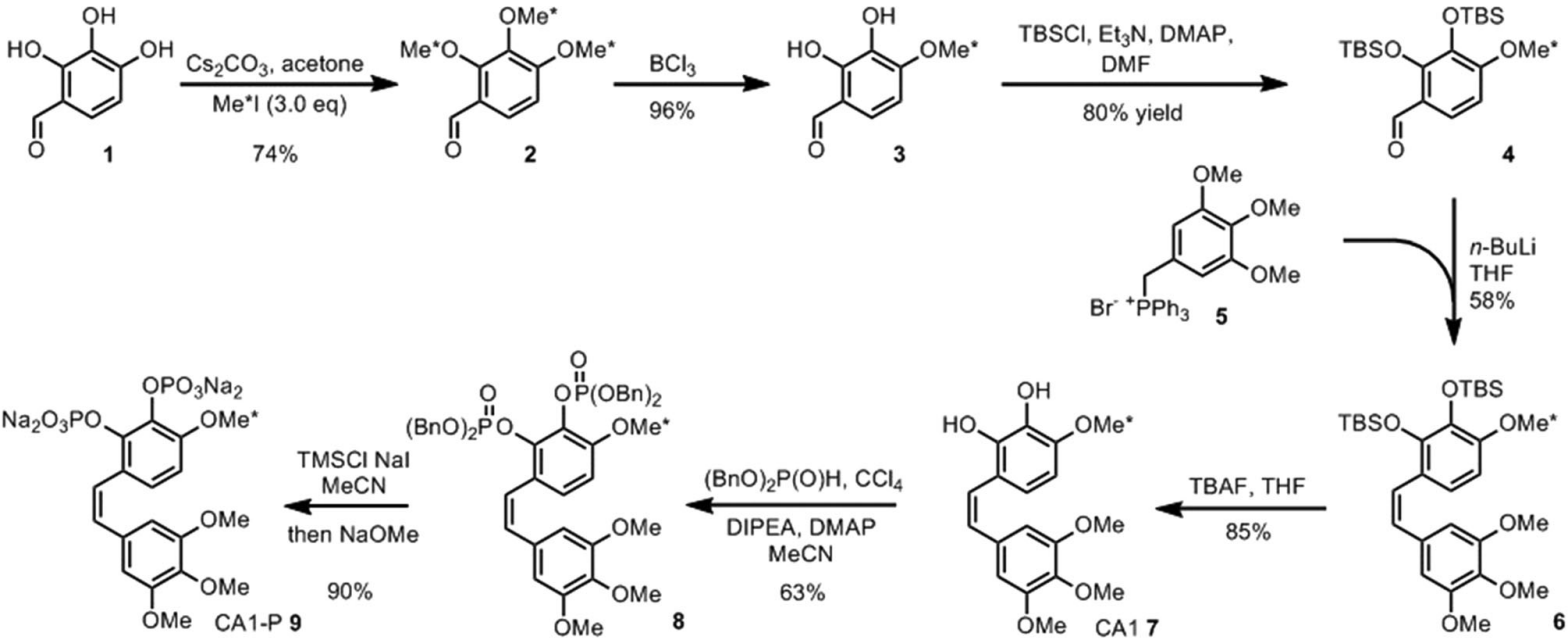
Synthetic route for combretastatin CA1‐P. * indicates the location of the ^13^CD_3_‐labeled methyl group. Bn, benzyl; DIPEA, *N,N*‐Diisopropylethylamine; DMAP, 4‐Dimethylaminopyridine; DMF, dimethylformamide; TBAF, tetra‐n‐butylammonium fluoride; TBS, tert‐butyldimethylsilyl; THF, tetrahydrofuran; TMSBr, Bromo(trimethyl)silane. (See Supporting Information for full details.)

The phosphorylated prodrug CA1‐P, was synthesized by adapting an existing synthetic procedure 20 to provide a ^13^C‐labeled and deuterated methyl group (^13^CD_3_) on the “B” ring of the molecule, as outlined in Figure 2. The synthetic procedure and associated analytical data are described in detail in the Supporting Information. A total of 160 mg ^13^CD_3_‐CA1‐P was dissolved in 175 μL 1:1 DMSO:D_2_O, including 14 mM Finland trityl radical (Oxford Instruments) and polarized for approximately 3 h before transfer to the injection system in deuterated HEPES buffer solution, as for glucose.

### MR Experiments

Each animal was placed in a supine position in a specially developed animal holder and located at the iso‐center of a 7 Tesla MRI scanner (Bruker Biospec 70/30, Avance III spectrometer, 120‐mm diameter bore, 660 mT/m gradient strength insert, Bruker Biospin MRI GmbH, Ettlingen, Germany). For evaluation of the intra‐arterial injection (IA) methodology, the hyperpolarized HEPES‐buffered pyruvate solution (∼80 mM for most of the animals) was administered directly to the tumor via the superior epigastric artery cannula using a custom injection system 19. Automated injection started immediately on receipt of the hyperpolarized substrate by the injector (ie, 4.5 s after opening of the solvent vessel valve on the HyperSense polarizer). The injector was initially set to flow to waste at 20 mL/min to fill the cannula dead‐space (0.8 mL; Fig. 1). Then, 20 µL at 10 mL/min was delivered to the rat (to overcome arterial blood pressure) followed by 180 µL at 1 mL/min to infuse the tumor with pyruvate. The total injected pyruvate volume was therefore 200 µL, resulting in an approximate dose to the whole animal of 5 mg/kg body weight. The injection protocol was chosen, so that the infused pyruvate was washed directly into the superior epigastric artery supplying the tumor via femoral artery blood flow. The estimated time from opening of the solvent vessel valve on the polarizer to hyperpolarized pyruvate entering the rat's vascular system was 7 to 8 s. The ^13^C‐metabolite signals were localized by a 20 mm ^13^C/^1^H surface coil (Bruker) above the tumor and 10 mm thick slice selection (20° flip angle (FA); sweep width (SW) = 50 ppm; time domain points (TD) = 256; repetition time (TR) = 1 s, number of excitations (NEX) = 1, number of repetitions (NR) = 180). A 6.7‐M ^13^C_1_‐acetate phantom was placed on top of the surface coil to provide a constant reference signal for frequency setting and assessment of reproducibility. To compare this method with the more usual systemic intravenous (IV) injection, a control group of rats bearing inguinal fat pad tumors was used, in which a contralateral femoral vein was cannulated to allow systemic IV infusion. In this case, the maximum permissible volume of 5 mL/kg body weight of the 80 mM pyruvate solution was infused via the cannulated femoral vein (equivalent to a pyruvate dose of 36 mg/kg body weight (ie, approximately 7 times higher than the whole body dose used for IA administration). Intravenous infusion was carried out with the same initial flow to waste (0.8 mL at 20 mL/min to fill the cannula dead‐space), followed by a 5 mL/kg infusion over 13 s, using the automated injection system described previously. As for the IA infusions, the estimated time from opening of the solvent vessel valve on the polarizer to hyperpolarized pyruvate entering the rat's vascular system was 7 to 8 s. In a subset of IA experiments, localization of the pyruvate/lactate signal was carried out using a spectral‐spatial (spsp) selective pulse 21, combined with an echo planar read‐out trajectory, to rapidly image pyruvate to lactate conversion in a single axial slice containing the tumor and surrounding tissue. The excitation frequencies were located at the ^13^C_1_ resonant frequencies for pyruvate, lactate, and acetate (field of view = 80 × 40 mm, 32 × 32, slice thickness = 12 mm, TE/TR = 18.42/1000 ms, nominal FA = 70^o^, NEX = 1, NR = 90).

To test the application of the method to a molecule with a shorter T_1_, ^13^C_u_‐glucose‐d7 (in vivo apparent T_1_ reported as 9 s 9) was polarized and infused IA as described previously at differing concentrations. The hyperpolarized signal was acquired using the surface coil described previously with a nonlocalized pulse‐acquired sequence (60 μs block pulse, 20° FA, SW = 200 ppm, TD = 2048, TR = 1 s, NEX = 1, NR = 100). Hyperpolarized ^13^C‐labeled CA1‐P was administered IA using the injection protocol described previously and data acquired using a nonlocalized spectroscopic acquisition with a broadband radiofrequency (RF) pulse (60 μs block pulse, 15° FA, SW = 200 ppm, TD = 2048, NEX = 1, NR = 80).

### Gadolinium Perfusion

Following IA infusion of hyperpolarized glucose, six of the nine animals used were rapidly imaged while administering a gadolinium‐based contrast agent via the tumor‐supplying artery, to investigate the perfusion of the tissue‐isolated tumors relative to surrounding normal tissue in a larger cohort than used for optical imaging. The same injection protocol as for the hyperpolarization experiments was used. A 5:1 diluted solution of gadopentetate dimeglumine (Magnevist, Bayer Schering Pharma AG, Berlin, Germany) was administered, and up to 100 FLASH images were acquired during and after the infusion (field of view ∼40 × 40 mm, 1 × 1 mm slice, 64 × 64, FA 30°, TR/TE 50.05/6 ms, NEX = 1, NR = 100, image acquired every 3.2 s).

### Data Processing

All hyperpolarized spectra were processed from the raw data using custom MATLAB software (MathWorks, Natick, MA, USA), to Fourier transform, phase, and baseline correct the spectra. Peaks of interest (eg, pyruvate/lactate, glucose) were integrated over the time course to produce ^13^C integral versus time curves. The forward pyruvate‐lactate conversion rate constant, k_pl_, was estimated using a precursor‐product relationship as previously described 22. Results are quoted as mean ± standard error of mean (SEM), unless otherwise stated. Where a linear regression line is fitted to the data, the Pearson correlation coefficient, *r*, and statistical significance, *p*, are quoted. The significance of differences between two groups was assessed in MATLAB using the student's two‐tailed t‐test, with equal variance for unpaired data, with *P* < 0.05 considered significant.

## RESULTS

### Perfusion of Tissue‐Isolated Tumors

**Figure 3:**
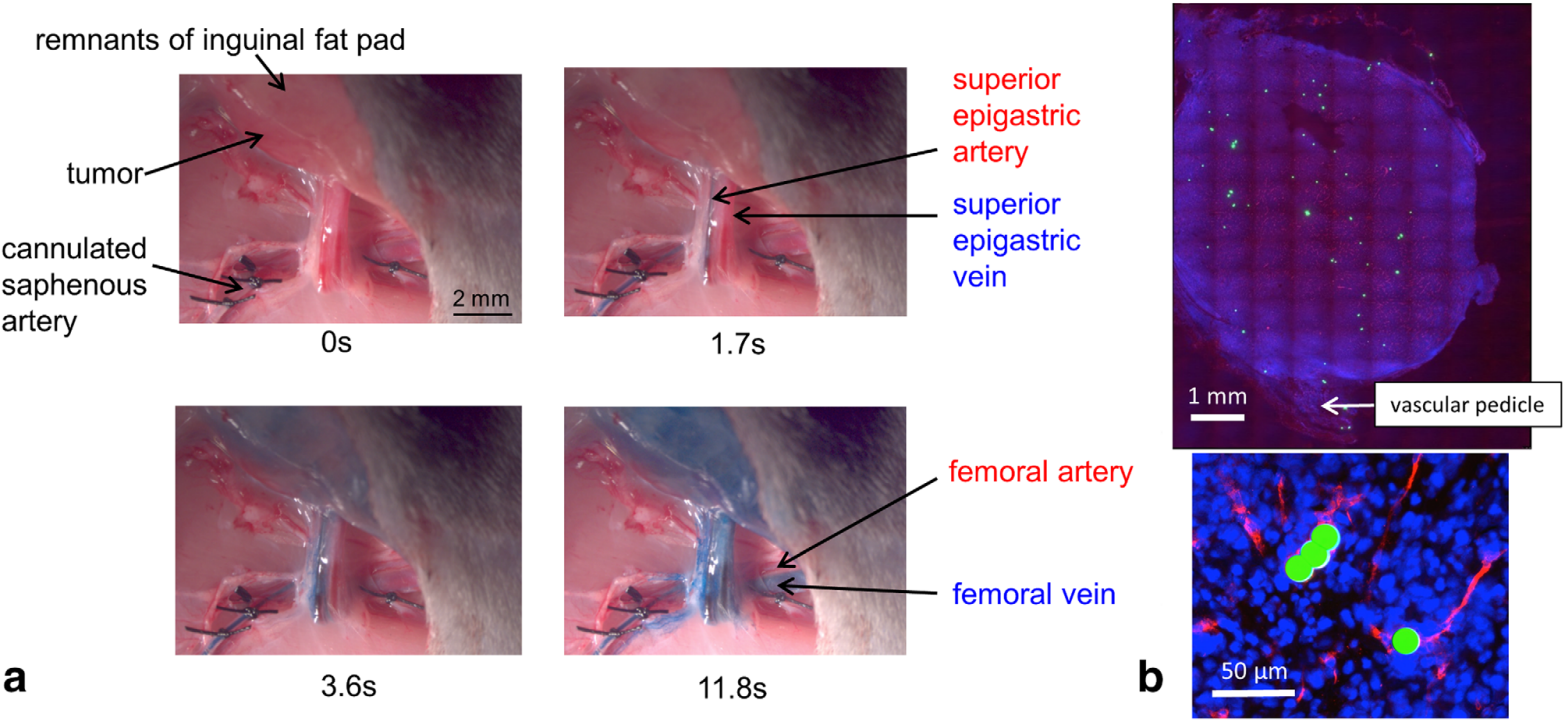
(**a**) Tumor implantation site in the right inguinal cleft at different times after the start of IA infusion of methylene blue into a male BDIX rat (see also Supporting Video S1). Note that the femoral artery remained clear of methylene blue during the infusion time. (**b**) Fluorescent microbeads (green) trapped in the tumor vasculature stained for CD31 (red) following IA infusion. Nuclear staining (DAPI; 4',6‐diamidino‐2‐phenylindole) is shown in blue.

Preliminary optical imaging experiments using methylene blue infusion via the cannulated saphenous artery established that an IA infusion rate of 1 mL/min effectively perfused the tumor and overlying fat without any observable perfusion of surrounding normal tissues or retrograde flow along the femoral artery (Fig. 3a and Supporting Video S1). Therefore, this flow rate was carried forward to the MR experiments. Fluorescent microbeads, infused at 1 mL/min via the same route, were distributed uniformly across tumor sections (Fig. 3b), indicating that the superior epigastric artery was the critical source of blood flow to the whole tumor microcirculation.

**Figure 4:**
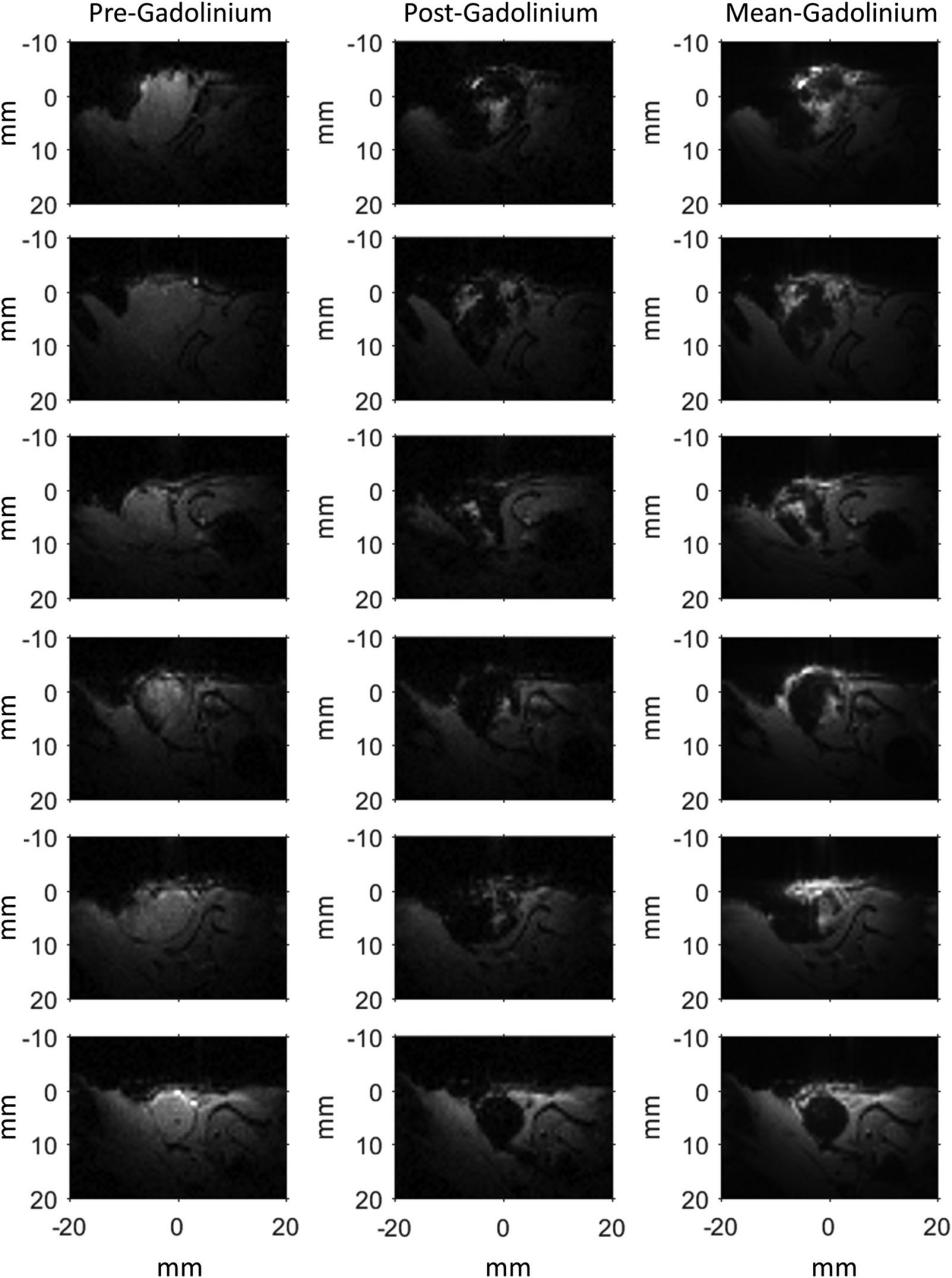
FLASH images acquired before and during IA infusion of one in five saline‐diluted Magnevist (gadolinium‐based contrast agent; FA 30°, TR/TE = 50.05/10.5 ms, 64 × 64, 40 × 40 mm). Magnevist was infused after completion of the hyperpolarized glucose experiments in six of the nine animals shown in Figure 6b; glucose concentrations, top to bottom, are 10, 20, 20, 20, 42, and 63 mM. Each row represents a single tumor. Images were acquired every 3.2 s, with the pre‐gadolinium images in the left column showing the last image acquired just before an observable change in image intensity (as judged by eye by S.R., M.P.) as a result of a high concentration of gadolinium, typically approximately 6 s after commencing the injection protocol at time = 0. This time included 4.5 s allocated to hyperpolarized sample transfer from the polarizer to the injection system. The post‐gadolinium images in the middle column are the images with the greatest change in image intensity within the tumor region (as judged by eye by S.R., M.P.) following the start of Magnevist infusion. These images occurred 9 to 13 s after the initial change in voxel intensity and are assumed to represent the highest concentration of gadolinium obtained in the tumor tissue. The right column (mean‐gadolinium) shows the mean of images acquired over 60 s, starting from the time point of the pre‐gadolinium (left side) image. The full time course of images for the tumor in the sixth row is shown in Supporting Video S2.

To support the optical imaging findings, a gadolinium‐based contrast agent was infused via the cannulated saphenous artery, as described previously, following the hyperpolarized glucose experiments. Single‐slice ^1^H gradient echo images were acquired every 3.2 s before and during gadolinium infusion. Figure 4 shows ^1^H FLASH images of tumors just before a change in the ^1^H image voxel intensity within the tumor region (denoted as “pre‐gadolinium”) and at the maximum decrease in ^1^H image voxel intensity within the tumor region (denoted as “post‐gadolinium”), assumed to represent the maximum tumor gadolinium concentration. Both time points were judged by eye by two independent experts: S.R. and M.P. In addition, the mean of all of the images acquired over 60 s from the time of the pre‐gadolinium image is shown for each tumor (denoted as “mean‐gadolinium”). The initial change in image intensity was typically first observed at approximately 9 s ‐ similar to that observed for hyperpolarized pyruvate and glucose, as described subsequently. Maximum gadolinium concentration occurred between 9 and 13 s after the first observed change. In some cases during infusion, the entire tumor region became darker, as a result of high gadolinium concentration substantially shortening the $T_{2}^{*}$, whereas the surrounding tissue was unchanged. In other cases, changes in the tumor were more heterogeneous; image intensity darkened or brightened depending on the local gadolinium concentration. This made quantitation of the perfusion changes problematic. However, the mean‐gadolinium images show that there were substantial intensity changes in tumor but not surrounding tissues over 60 s of Magnevist infusion, during which time gadolinium uptake is mostly a reflection of blood flow rate and vascular permeability (Fig. 4 and Supporting Video S2). This supports effective perfusion of tumor tissue via the saphenous artery, confirming preliminary data from optical imaging (Fig. 3). Furthermore, the data suggest that pre‐infusion of glucose, at the concentrations used, had little or no lasting influence on tumor vascular function, consistent with previously published data 23. At later time points than shown in Figure 4, voxel intensity changes were observed in the surrounding tissue, as the gadolinium was systemically distributed (see Supporting Video S2).

### Signal Enhancement by IA Administration: Pyruvate Metabolism

Mean arterial blood pressure for cannulated rats immediately prior to IA infusion of pyruvate and slice‐selected scanning was 76 ± 2 mmHg (n = 9) compared with 80 ± 8 mmHg (n = 5) for the IV cannulated group. The equivalent breathing rates were 54 ± 2 and 57 ± 7 breaths per minute, respectively. These values were similar and not significantly different between the two groups (*P* = 0.49 and 0.51 for blood pressure and breathing rate, respectively), indicating a similar physiological status.

**Figure 5:**
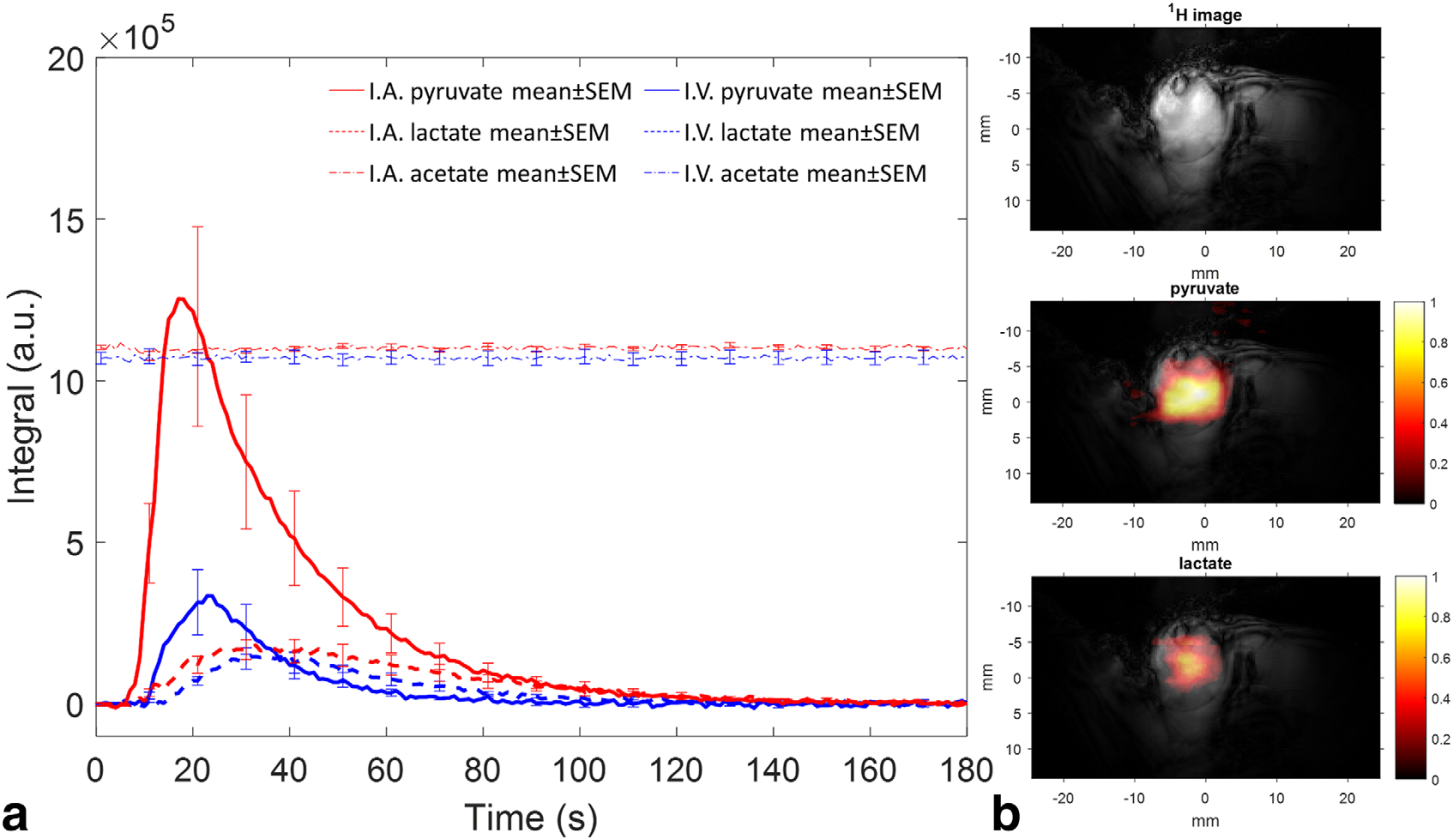
(**a**) Hyperpolarized pyruvate and lactate tumor time courses for IA administration (red traces; n = 9: 7 male; 2 female) and IV injection (blue traces; n = 5 male). Male rats weighed 299 ± 21 g for IA and 297 ± 18 g for IV (mean ± SD); female rats weighed 174 and 181 g. Data were acquired every 1 s. Lines show mean values; error bars are SEM, shown at 10 s intervals for clarity. Integrals of signal intensity are measured in arbitrary units (a.u.). Acetate integrals are offset from their true position by 1e6 for clarity. Time zero is the start of sample dissolution, corresponding to the opening of the solvent vessel valve on the HyperSense polarizer. Based on known cannula dead spaces and infusion rates, the approximate time for hyperpolarized pyruvate to enter the vascular system was 7 to 8 s from time 0 s, including 4.5 s to allow the sample to transit from the polarizer to the injection system, after which the injection protocol commenced. (**b**) Example of a composite spectral‐spatial‐EPI image. Top image shows the ^1^H structural image; middle and bottom images show the^13^C pyruvate and lactate images superimposed on the ^1^H structural image, respectively. ^13^C images consist of data from 40 axial images acquired every 1 s and are normalized to the maximum intensity of the pyruvate signal.

The mean areas under the pyruvate and lactate signal time course (AUC) were compared for the direct arterial versus the systemic venous infusion routes (Fig. 5a). For an 80‐mM pyruvate infusion, the mean IA AUC was 4.6 and 1.6 times greater than the IV AUC for pyruvate and lactate, respectively. The tumor weights for each group were not significantly different (IA tumor = 1.44 ± 0.24 g (n = 9) and IV tumor = 0.92 ± 0.20 g (n=5); *P* = 0.17). The mean IA dose per kilogram of rat body weight was 5.4 ± 0.5 mg/kg (mean rat weight 272 ± 19 g; 1.4 mg pyruvate administered; n = 9), compared with a mean IV dose of 36 mg/kg (mean rat weight 297 ± 8 g). Therefore, the actual signal enhancement for the IA injected pyruvate was 33‐fold (4.6*36/5). The IA pyruvate signal was first observed above the noise level after 9.2 ± 0.6 s, with a signal maximum occurring at 15.7 ± 1.1 s. This compares to 10.6 ± 0.7 s and 22.4 ± 1.1 s for the IV times, respectively. Correspondingly, the IA lactate signal was first observed above the noise level at 11.6 ± 0.8 s, with a signal maximum at 30.6 ± 1.4 s. This compares to 14.6 ± 0.5 s and 34.8 ± 1.4 s for the IV times, respectively. A student's t‐test showed a statistically significant shorter rise time (time from first signal observation to signal maximum) for IA versus IV tumors for pyruvate (*P* = 0.007), but not for lactate (*P* = 0.51).

Spectral‐spatial pulse echo‐planar imaging (EPI) acquisitions in separate animals confirmed results for methylene blue and gadolinium perfusion, showing that most of the IA pyruvate initially perfused only the tumor rather than the surrounding normal tissue, with a complementary lactate signal in tumor tissue (Fig. 5b).

Applying a precursor‐product model 22 to the slice localized data, the rate constant for the conversion of pyruvate to lactate, *k*_*pl*_, was 0.020 ± 0.004 s^‐1^ for IA injections (n = 9) compared with 0.052 ± 0.009 s^‐1^ for IV injections (n = 5). These values were significantly different (*p* = 0.022). Two further hyperpolarized pyruvate IA injections were performed, in two separate male animals, at lower doses. The estimated *k*_*pl*_ for these were 0.035 and 0.023 s^‐1^ for 10 and 20 mM of injected pyruvate concentrations, respectively.

### IA Administration of a Substrate with Short T_1_: Glucose Metabolism

**Figure 6:**
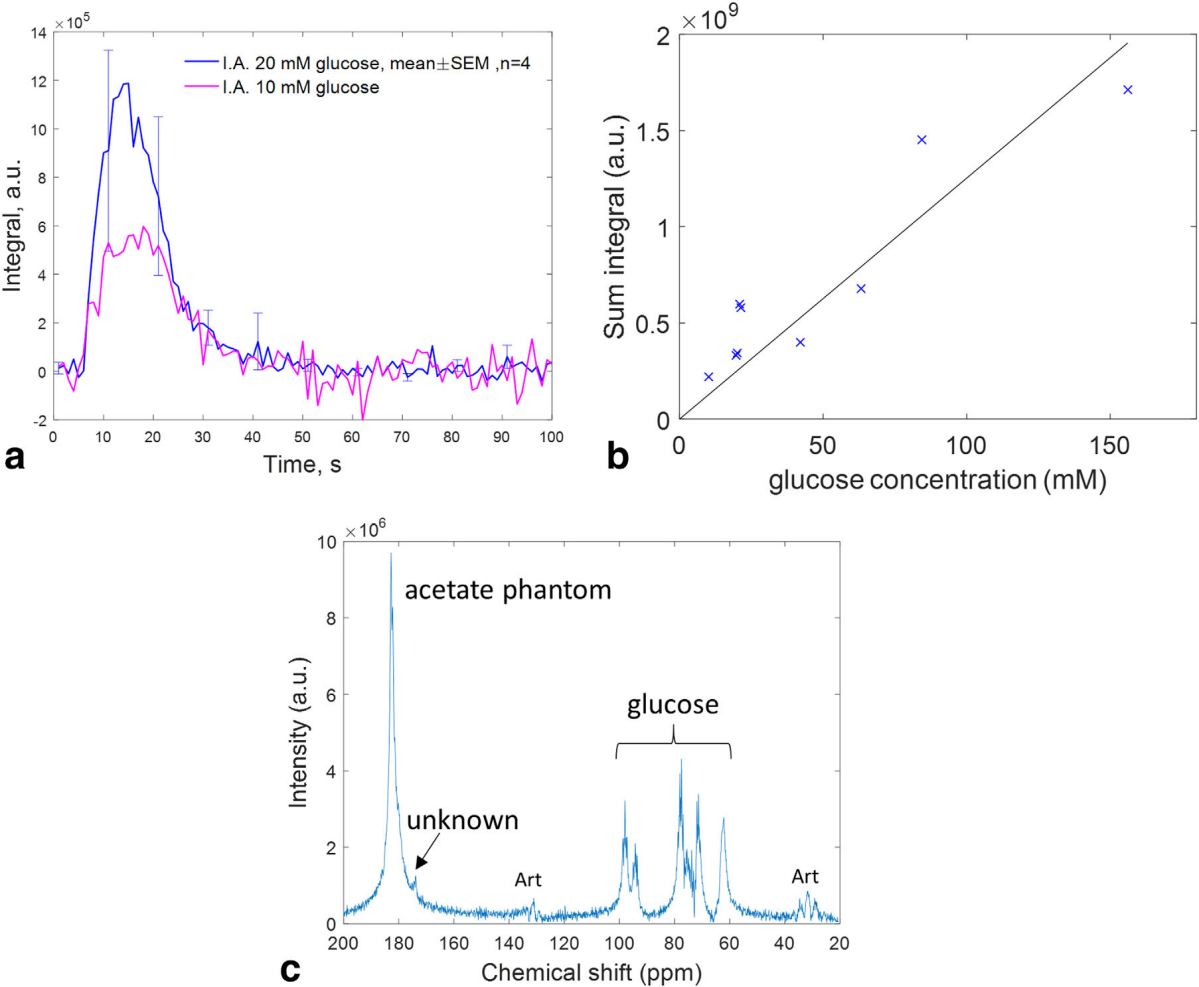
(**a**) Hyperpolarized ^13^C_u_‐glucose‐d7 time course for IA administration. Plot shows mean ± SEM trace (n = 4) for 20 mM solution (error bars are shown at 10‐s intervals for clarity) and a single trace for 10‐mM solution (all male). Integrals of signal intensity are measured in arbitrary units (a.u.). Data were acquired every 1 s. Time zero is the start of sample dissolution, corresponding to opening of the solvent vessel valve on the HyperSense polarizer. (**b**) The AUC of hyperpolarized glucose time courses versus injected glucose concentration; the AUC is measured in arbitrary units (a.u.). Data include the examples shown in (**a**). Each point represents a single animal (n = 9 male rats weighing 306 ± 27 g (mean ± SD)). (**c**) Composite spectrum from 180 time points after injecting 63 mM hyperpolarized ^13^C_u_‐glucose‐d7 in a single tumor. Peak at 173.9 ppm assigned to glutamate. Spectrum referenced to ^13^C_1_‐glucose peak at 98 ppm. Art, artifact.

Hyperpolarized glucose was used as a readily available test molecule that possesses a short T_1_, similar to the proposed ^13^C‐labeled CA1‐P to optimize the acquisition procedure. Following IA administration of hyperpolarized glucose, the time‐course signal could be first detected at 8.1 ± 0.5 s with a maximum signal at 15.6 ± 0.4 s (Fig. 6a). Additionally, there was a strong correlation (*r* = 0.93, *p* < 0.001) between glucose concentration and glucose signal between 10 and 156 mM (Fig. 6b). No lactate peak was observed. However, in a subset of the in vivo experiments (3 of 9 shown in Fig. 6b), an additional unknown peak was observed at 9 ppm up field from the acetate phantom, at 174 ppm, that was tentatively assigned to glutamate. An example is shown in Figure 6c. There was no relationship to glucose dose, as two of the three tumors with the additional peak received glucose at 20 mM, and the third received 63 mM. We also carried out further experiments using the same single‐slice spectral‐spatial pulse as used for EPI imaging of pyruvate (Fig. 5b), coupled with a one‐dimensional spectral acquisition. The spectral excitation frequencies were set to those for glucose and lactate (with a 50‐ppm spectral acquisition window), in an attempt to detect lactate production. However, no lactate peak was observed (n = 5; data not shown).

### IA Administration of a Hyperpolarized Drug: Combretastatin

**Figure 7:**
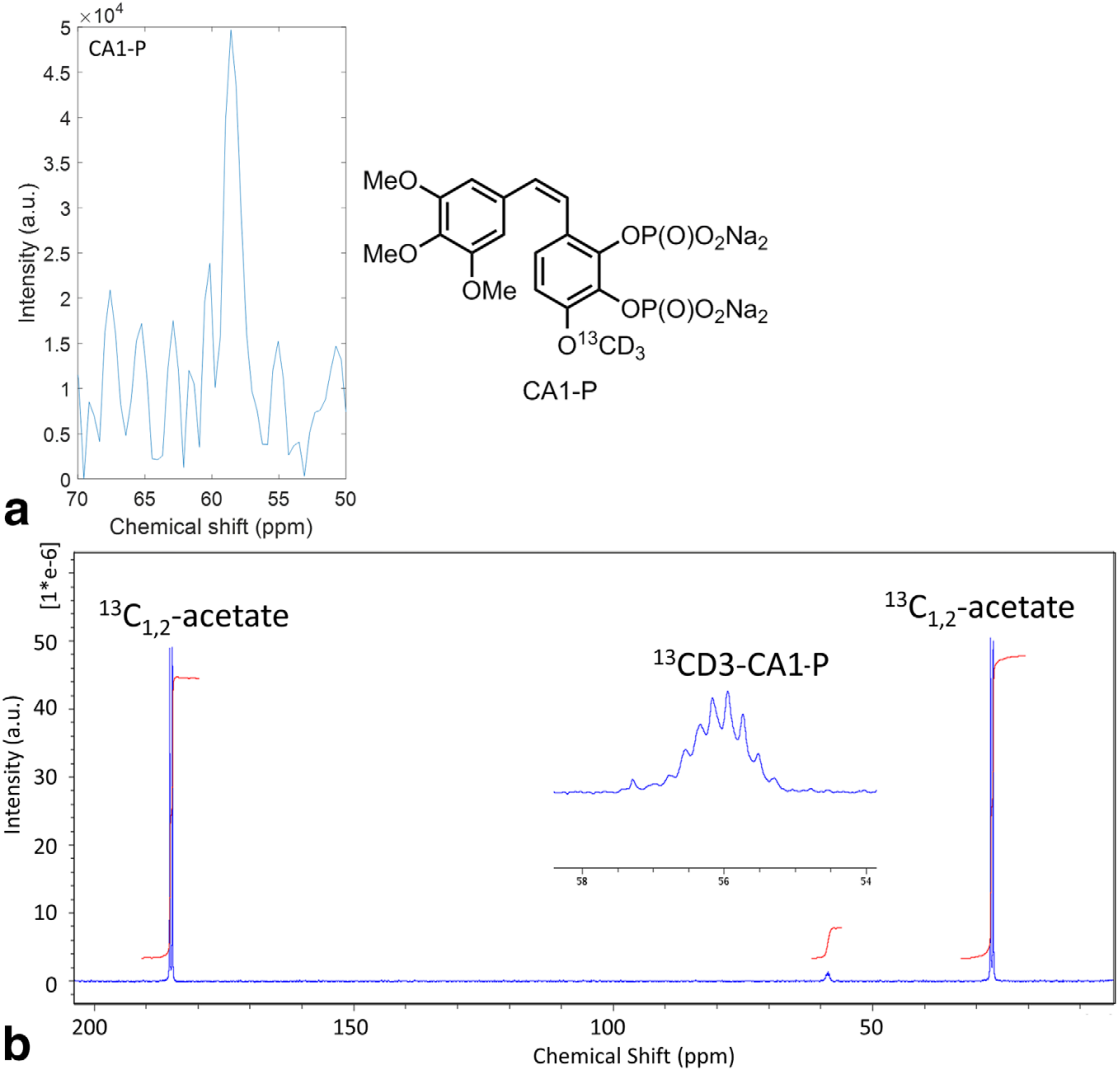
(**a**): Hyperpolarized in vivo tumor signal from CA1‐P (chemical structure shown) following IA infusion in a single 233 g male rat (FA = 25°, TR = 1 s, SW = 200 ppm, TD = 512, NEX = 80); ^13^C labeled at the 15 position. (**b**) ^13^C{^1^H}‐inverse gated spectrum of 15 mM CA1‐P dissolved in H_2_O (20% D_2_O) with 80 mM ^13^C_1,2_‐acetate as reference peaks. Inset shows expanded part of the spectrum containing CA1‐P signal. Spectrum acquired on a 9.4 T Bruker Avance III spectrometer (FA = 90°, TR = 100 s, SW = 200 ppm, TD = 8062, NEX = 512).

The signal from hyperpolarized ^13^C‐labeled CA1‐P was too small to be observed in the individual time‐course data, but was clearly visible at 58 ppm when all of the time‐course data were summed (Fig. 7a), matching a previously measured spectrum of CA1‐P acquired for an aqueous sample on a 9.4 T spectrometer (Fig. 7b).

## DISCUSSION

Direct infusion into a tumor‐supplying artery enabled rapid perfusion of the tumor tissue with hyperpolarized substrate and a reduced systemic dose compared with that from conventional IV injection. Optical and MR imaging showed that IA administered substrates/contrast agents almost exclusively perfused the tumor tissue on initial injection.

The enhanced signal level obtained with this technique (more than 30‐fold) allows for either reduced administered dose or use of substrates with lower polarization than the commonly used substrate, pyruvate. Tumors used for IV infusion of pyruvate were approximately 36% smaller, by mass, than those used for IA infusion. Although not a statistically significant difference, an increased signal intensity would have been expected in the IV infused tumors had they been larger. However, this clearly cannot account for the more than 30‐fold pyruvate signal enhancement obtained for IA administration. As expected, the rise time for pyruvate was significantly shorter for IA administration than IV administration, indicating faster delivery to the tumor. However, the lactate rise time was unaffected by the route of administration, suggesting that the increased pyruvate delivery had little effect on its metabolism.

The significantly enhanced pyruvate signal in the tumor for IA compared with IV administration indicates rapid extraction of pyruvate from the circulation into the tumor tissue on first pass. The circulation time in rats at the body weights used in our experiments was approximately 7 s 24, so the recirculation of hyperpolarized pyruvate would have approximately coincided with the end of the infusion period. In addition, any pyruvate not extracted by the tumor tissue on first pass would have been largely diluted in the systemic circulation, while also rapidly losing signal. Taken together, these considerations suggest that recirculation of pyruvate had little influence on the observed pyruvate kinetics in the tumor.

An injected concentration of 80 mM pyruvate, used for most animals in this study, is within the range typically used for IV administration of pyruvate in hyperpolarization experiments. In our IV case, this equated to 36 mg/kg or 0.4 mmol/kg total dose. Janich et al 25 reported partial tissue saturation in rat liver, kidney, and heart by pyruvate at 0.2 to 0.4 mmol/kg pyruvate. Our estimated *k*_*pl*_ values decreased with an increasing IA dose (between 10 and 80 mM in 200 µL) and were lower than the *k*_*pl*_ estimated from IV administration of pyruvate, suggesting saturation of tumor lactate dehydrogenase, at least in the IA case. This would be expected based on the large total doses delivered directly to the tumor, and would explain why the observed increase in the pyruvate signal for IA versus IV infusion was not matched by a corresponding increase in the lactate signal (Fig. 5). However, because *k*_*pl*_ represents the fractional turnover of pyruvate, rather than the absolute rate of pyruvate to lactate production, it is difficult to draw any firm conclusions regarding enzyme kinetics in the face of different concentrations of administered pyruvate. Furthermore, the relative amplitude of the lactate time‐course signal also depends on the size of the endogenous lactate pool, as there is a limit imposed on the observable ^13^C lactate signal due to the lactate pool's ability to retain ^13^C spins 26. However, the current model provides a means of accurately titrating administered doses in a more controlled manner than for systemic administration, either to avoid or specifically investigate processes such as tissue saturation and toxicity. In addition, accurate knowledge of the concentration of administered substances in the tumor arterial blood supply provides the opportunity for accurate estimation of blood‐tissue exchange of substrates and a ready arterial input function for modeling purposes, at least for the first few seconds of infusion.

The concentrations of IA administered glucose used in our study were at the upper range of physiological blood glucose concentrations. Observation of glucose metabolism was limited to a small subset of tumors, despite trialing different MR acquisitions methods (eg, slice selection, spsp RF excitation) for improved signal detection. Although a tentative assignment, the observation of glucose to glutamate conversion, but not lactate, is surprising given that tumors are generally characterized by lactate production under aerobic as well as anaerobic conditions. However, high glucose exposure has been found to increase cellular glutamate (via α‐ketoglutarate) in a number of different cell types in vitro 27. Further investigations would be needed to positively assign the peak that we identified in a subset of the glucose experiments to glutamate.

The additional metabolic steps from glucose to lactate production or reduced active transport of glucose into cells compared with pyruvate, coupled with a short T_1_ for glucose, may explain the lack of a tumor lactate signal, in contrast to the readily observable lactate signal on administration of hyperpolarized pyruvate. However, Rodrigues et al reported glucose‐to‐lactate conversion in the mouse T cell lymphoma (EL4) and Lewis lung carcinoma following systemic IV administration of hyperpolarized glucose 9. It is possible that glucose metabolism is highly dependent on the tumor model used. Rodrigues et al reported a steady‐state lactate concentration of 20.7 ± 3.0 µmol/g for the EL4 tumor, whereas we previously reported a concentration of 3.0 ± 0.2 µmol/g for the P22 tumor 28. This lower concentration is consistent with the P22 tumor being relatively well perfused and oxygenated 29, 30. A smaller lactate pool in the P22 tumor could also contribute to the lack of observable lactate signal on administration of hyperpolarized glucose. It is possible that further improvements in polarization level or experimental sensitivity could enable improved MR detection of glucose metabolism.

Despite the desirability of imaging drug metabolism directly, only a drug's indirect effect on metabolism has previously been shown using hyperpolarized MRS 31. Hyperpolarized drug detection is hampered by short T_1_ relaxation times that are on the order 1 to 10 s 6. Typical drug molecules possess molecular weights of a few hundred Daltons that can result in molecular rotational correlation times that are ideally suited to provide an efficient relaxation pathway. By a judicious labeling strategy (ie, deuteration and ^13^C label location), T_1_ relaxation time can be lengthened to the point where it is feasible to administer hyperpolarized drugs without too much signal loss. Relatively large amounts of CA1‐P were needed in the current study to observe an in vivo signal, and further work is clearly required to improve the polarization of these molecules. The pharmacokinetics of CA1‐P and its close analogues suggest that its metabolism is rapid 32. However, its metabolic conversion may still be slow compared with hyperpolarized signal lifetime. Therefore, the lack of any observable daughter metabolite in the MR spectrum could be the result of a combination of relatively slow metabolism and low level of hyperpolarization. A limitation on the use of hyperpolarization for studying drug metabolism is undoubtedly its restricted use to drugs with rapid metabolism, compatible with the lifetime of the hyperpolarized signal. However, our method shows that it is possible to observe hyperpolarized drugs in vivo without the requirement to administer a large systemic dose. The method has the potential to directly monitor drug distribution and metabolism kinetics in tumors, with the possibility of observing unforeseen side reactions.

## CONCLUSIONS

Here we report a method that enables expansion of the range of observable hyperpolarization targets for tumor studies to include those with lower levels of polarized signal, shorter T_1_ relaxation times, and those whose systemic toxicity is prohibitive if administered intravenously. Although we envisage the main utility of IA administration to be for preclinical studies of tumor energy and drug metabolism, its use in humans is theoretically possible in situations in which the IA route can be used for chemotherapy, such as during treatment of colorectal cancer liver metastases. Hitherto, hyperpolarized MRS/MRI has been used to observe indirectly the effect of drug treatment on tumor metabolism. For the first time there is the possibility of directly observing a drug molecule in vivo by ^13^C MRS.

## Supporting information

Additional Supporting Information may be found in the online version of this article

Supporting Information

**Video S1**. Tumor implantation site during perfusion with methylene blue.

**Video S2**. FLASH images acquired every 3.2 s (30° FA, TR/TE 50.05/10.5 ms, 64 × 64, 40 × 40 mm during of Injection of 1 in 5 saline‐diluted gadolinium (Magnevist) using the same injection protocol as for hyperpolarized experiments. The video is for the same animals as for the sixth row images in Figure 4.
