# Supplementary material for: Direct arterial injection of hyperpolarized 13C‐labeled substrates into rat tumors for rapid MR detection of metabolism with minimal substrate dilution
Source: Magn Reson Med. 2017 Feb 12;78(6):2116–26. doi: 10.1002/mrm.26628 (PMC5697693; doi:10.1002/mrm.26628)
Supplement: Supplementary file 1 — Supporting Information [file MRM-78-2116-s001.docx]

**[2,3,4-^13^CD_3_]-Trimethoxybenzaldehyde 2**

^13^CD_3_I (2 mL, 32.0 mmol) was added to a 0.5 M solution of 2,3,4-trihydroxybenzaldehyde **1** (1.5 g, 9.7 mmol) and Cs_2_CO_3_ (12.6 g, 39.0 mmol) in dry acetone at rt. The reaction mixture was heated at reflux for 3 h then allowed to cool to rt. The solvent was evaporated and the residue partitioned between water (100 mL) and ethyl acetate (100 mL). The aqueous phase was extracted with ethyl acetate (3 × 50 mL) and the combined organic phases were washed with brine (20 mL), dried over MgSO_4_, filtered and the solvent evaporated. The crude material was purified using flash column chromatography on silica gel, eluting with 0–50% EtOAc in petroleum ether (40-60) to give the title compound **2** as an oil (1.5 g, 74%); R*_f_* 0.35 [petroleum ether (40-60)–EtOAc (90:10)]; *ν*_max_ (film)/cm^-1^ 2857, 2067, 1683, 1586, 1489, 1445; ^1^H NMR (400 MHz, CDCl_3_) *δ* 10.23 (1H, s, C*H*O), 7.59 (1H, d, *J* 9.0 Hz, ArC*H*), 6.74 (1H, d, *J* 9.0 Hz, ArC*H*); ^13^C NMR (101 MHz, CDCl_3_) *δ* 188.8 (*C*H), 159.2 (*C*), 156.8 (*C*), 141.5 (*C*), 124.1 (*C*H), 123.2 (*C*), 107.4 (*C*H, d *J* 5.5 Hz), 61.4 (sept, *J* 22.5 Hz, ^13^*C*D_3_), 60.0 (sept, *J* 22.5 Hz, ^13^*C*D_3_), 55.3 (sept, *J* 22.5 Hz, ^13^*C*D_3_); *m/z* (TOF ES^+^) 209.1474 (100%, MH^+^_._ C_7_^13^C_3_H_4_D_9_O_4_ requires 209.1479).

**2,3-Dihydroxy-[4-^13^CD_3_]-methoxybenzaldehyde 3**

BCl_3_ (7.9 mL, 7.9 mmol. 1.0 M solution in CH_2_Cl_2_) was added dropwise at rt to a 0.15 M solution of ether **2** (1.5 g, 7.2 mmol) in dry CH_2_Cl_2_ (15 mL). The reaction was stirred for 2 h and then BCl_3_ (7.9 mL, 7.9 mmol, 1.0 M solution in CH_2_Cl_2_) was added. The reaction was stirred for 16 h and then saturated aqueous NaHCO_3_ (150 mL) was carefully added. The solution was acidified with 3 M HCl (150 mL) and then extracted with EtOAc (3 × 100 mL). The combined organic phases were washed with brine (30 mL), dried over MgSO_4_, filtered and the solvent evaporated. The crude material was recrystallized from EtOAc–petroleum ether (40-60) (1:1) to give the title compound **3** as a solid (1.2 g, 96%). R*_f_* 0.15 [petroleum ether (40-60)–EtOAc (80:20)]; *ν*_max_ (film)/cm^-1^ 3369, 1652, 1630, 1506, 1471; ^1^H NMR (400 MHz, CDCl_3_) *δ* 11.11 (1H, s, C*H*O), 9.74 (1H, s, O*H*), 7.13 (1H, d, *J* 8.5 Hz, ArC*H*), 6.61 (1H, d, *J* 8.5 Hz, ArC*H*), 5.58 (1H, s, O*H*); ^13^C NMR (101 MHz, CDCl_3_) *δ* 195.2 (*C*H), 153.0 (*C*), 149.0 (*C*), 133.0 (d, *J* 3.0 Hz, *C*), 126.1 (*C*H), 116.0 (*C*), 103.6 (d, *J* 4.5 Hz, *C*H), 55.5 (sept, *J* 22.5 Hz, ^13^*C*D_3_); *m*/*z* (TOF ES^+^) 173.0716 (100%, MH^+^. C_7_^13^CH_6_D_3_O_4_ requires 173.0723).

**2,3-*bis*-[(*tert*-Butyldimethylsilyl)oxy]-[4-^13^CD_3_]-methoxybenzaldehyde 4**

Et_3_N (3.9 mL, 28.0 mmol) and DMAP (171 mg, 1.4 mmol) were added to a 0.5 M solution of diol **3** (1.19 g, 0.69 mmol) in dry DMF at rt, followed by a solution of TBDMSCl in DMF (4.2 g, 28.0 mmol). The reaction was stirred at rt for 16 h, then water (200 mL) and ethyl acetate (200 mL) were added. The phases were separated and the aqueous phase extracted with ethyl acetate (3 × 100 mL). The combined organic phases were washed with 10% LiCl (100 mL), brine (100 mL), dried over MgSO_4_, filtered and the solvent was evaporated. The crude material was purified using flash column chromatography of silica gel, eluting with 0–5% EtOAc in petroleum ether (40-60) to give the title compound **4** as an oil (2.2 g, 80%). R*_f_* 0.25 [petroleum ether (40-60)–EtOAc (95:5)]; *ν*_max_ (film)/cm^-1^ 2935, 2858, 1656, 1583, 1494, 1442; ^1^H NMR (400 MHz, CDCl_3_) *δ* 10.23 (1H, s, C*H*O), 7.49 (1H, d, *J* 9.0 Hz, ArC*H*), 6.62 (1H, d, *J* 9.0 Hz, ArC*H*), 1.05 [9H, s, (C*H*_3_)_3_C], 1.00 [9H, s, (C*H*_3_)_3_C], 0.14 (12H, s, 4 × C*H*_3_); ^13^C NMR (101 MHz, CDCl_3_) *δ* 189.3 (*C*H), 157.5 (*C*), 151.0 (*C*), 136.7 (*C*), 123.3 (*C*), 121.4 (*C*H), 105.4 (d, *J* 5.5 Hz, *C*H), 54.4 (sept, *J* 22.5 Hz, ^13^*C*D_3_), 26.2 [(*C*H_3_)_3_C], 26.0 [(*C*H_3_)_3_C], 18.8 [(CH_3_)_3_*C*], 18.6 [(CH_3_)_3_*C*], –3.9 (4 × *C*H_3_); *m*/*z* (TOF ES^+^) 401.2464 (100%, MH^+^. C_19_^13^CH_34_D_3_O_4_Si_2_, requires 401.2452).

**(*Z*)-2-(2,3-Bis[{*tert*-butyldimethylsilyl}oxy]-4-[^13^CD_3_]-methoxyphenyl)-1-(3,4,5-trimethoxyphenyl)ethene 6**

*n*-BuLi (1.9 mL, 3.9 mmol, 2.0 M in hexanes) was added to a 0.25 M solution of 3,4,5-trimethoxybenzyltriphenylphosphonium bromide^[[1]](#footnote-1)^ **5** (1.9 g, 3.6 mmol) in dry THF at –20 ^o^C. The reaction was stirred for 30 min and then aldehyde **4** (1.2 g, 3.0 mmol) was added. The reaction was warmed to rt over 2 h and then water (100 mL) and ethyl acetate (100 mL) were added. The phases were separated and the aqueous phase extracted with ethyl acetate (50 mL × 3). The combined organic phases were washed with brine (30 mL), dried over MgSO_4_, filtered and the solvent was evaporated. The crude material was purified using flash column chromatography on silica gel, eluting with 0–25% EtOAc in petroleum ether (40-60) to give a mixture of *cis* and *trans* product (1.52 g, 85%). The *cis-*(*Z*)-isomer was isolated by recrystallization from ethanol to give the title compound **6** as a crystalline solid (1.1 g, 58%). R*_f_* 0.25 [petroleum ether (40-60)–EtOAc (90:10)]; *ν*_max_ (film)/cm^-1^ 2927, 2854, 1576, 1498, 1439; ^1^H NMR (400 MHz, CDCl_3_) *δ* 6.92 (1H, d, *J* 8.5 Hz, ArC*H*), 6.63 (2H, s, 2 × ArC*H*), 6.60 (1H, d, *J* 12.0 Hz, C*H*=CH), 6.37 (1H, d, *J* 8.5 Hz, ArC*H*), 6.38 (1H, d, *J* 12.0 Hz, C*H*=CH), 3.84 (3H, s, OC*H*_3_), 3.68 (6H, s, 2 × OC*H*_3_), 1.05 [9H, s, (C*H*_3_)_3_C], 1.01 [9H, s, (C*H*_3_)_3_C], 0.20 (6H, s, 4 × C*H*_3_), 0.11 (6H, s, 6 × C*H*_3_); ^13^C NMR (101 MHz, CDCl_3_) *δ* 152.7 (*C*), 151.7 (*C*), 146.2 (*C*), 136.9 (*C*), 136.8 (*C*, d, *J* 3.0 Hz), 132.8 (*C*), 127.6 (*C*H), 127.3 (*C*H), 123.1 (*C*), 122.2 (*C*H), 105.9 (*C*H), 104.1 (*C*H, d, *J* 4.6 Hz), 60.9 (*C*H_3_), 55.8 (2 × *C*H_3_), 54.1 (sept, *J* 21.6 Hz, ^13^*C*D_3_), 26.4 [(*C*H_3_)_3_C], 26.1 [(*C*H_3_)_3_C], 18.7 [(CH_3_)_3_*C*], 18.6 [(CH_3_)_3_*C*], -3.3 (2 × *C*H_3_), -3.9 (2 × *C*H_3_); *m*/*z* (TOF ES^+^) 565.3309 (100%, MH^+^. C_29_^13^CH_46_D_3_O_6_Si_2_, requires 565.3290.

**3-[^13^CD_3_]-Methoxy-6-[(1*Z*)-2-(3,4,5-trimethoxyphenyl)ethenyl]-1,2-benzenediol CA1 7**

TBAF (10.6 mL, 10.6 mmol, 1.0 M in THF) was added dropwise to a 0.25 M solution of **6** (1.5 g, 2.7 mmol) in THF at rt. The reaction was stirred for 30 min and then water (100 mL) and ethyl acetate (100 mL) were added. The phases were separated and the aqueous phase extracted with ethyl acetate (3 × 50 mL). The combined organic phases were washed with brine, dried over MgSO_4_, filtered and the solvent was evaporated. The crude material was purified using flash column chromatography on silica gel eluting with 20% EtOAc in petroleum ether (40-60) to give **7** as a solid (880 mg, 98%). R*_f_* 0.40 [petroleum ether (40-60)–EtOAc (50:50)]; *ν*_max_ (film)/cm^-1^ 3415, 2934, 1623, 1579, 1506, 1462; ^1^H NMR (400 MHz, CDCl_3_) *δ* 6.76 (1H, d, *J* 8.5 Hz, ArC*H*), 6.60 (1H, d, *J* 12.0 Hz, HC=C*H*), 6.53 (2H, s, 2 × ArC*H*), 6.53 (1H, d, *J* 12.0 Hz, HC=C*H*), 6.38 (1H, d, *J* 8.5 Hz, ArC*H*), 5.50 (2H, br s, 2 × O*H*), 3.83 (3H, s, OC*H*_3_), 3.67 (6H, s, 2 × OC*H*_3_); ^13^C NMR (101 MHz, CDCl_3_) *δ* 152.8 (*C*), 146.4 (*C*), 141.7 (*C*), 137.3 (*C*), 132.6 (*C*), 132.5 (*C*), 130.2 (*C*H), 124.1 (*C*H), 120.3 (*C*H), 117.9 (*C*), 106.0 (*C*), 102.9 (d, *J* 4.5 Hz, *C*), 60.9 (O*C*H_3_), 55.9 (2 × O*C*H_3_), 55.4 (sept, *J* 22.5 Hz, ^13^CD_3_); *m*/*z* (TOF ES^+^) 337.1576 (100%, MH^+^. C_17_^13^CH_18_D_3_O_6_ requires 337.1560.

**(*Z*)-2-(2,3-*Bis*[{*bis*(benzyloxy)phosphinoyl}oxy]-4-[^13^CD_3_]methoxyphenyl)-1-(3,4,5-trimethoxyphenyl)ethene 8**

CCl_4_ (5 mL, 52.0 mmol), DIPEA (1.8 mL, 4.0 mmol) and DMAP (64 mg, 0.5 mmol) were added sequentially to a 0.25 M solution of CA1 **7** (850 mg, 2.5 mmol) in dry MeCN at –20 C. The reaction mixture was stirred for 5 min and then freshly prepared dibenzyl phosphite (2.7 g, 10.4 mmol) was added. The reaction was warmed to rt over 2 h and then a 0.5 M solution of KH_2_PO_4_ (10 mL) added. The phases were separated and then aqueous phase extracted with ethyl acetate (3 × 50 mL). The combined organic phases were washed with brine (30 mL), dried over MgSO_4_, filtered and the solvent was evaporated. The crude material was purified using flash column chromatography on silica gel, eluting with 0–50% EtOAc in petroleum ether (40-60) to give **8** as an oil (1.4 g, 63%). R*_f_* 0.20 [petroleum ether (40-60)–EtOAc (50:50)]; *ν*_max_ (film)/cm^-1^ 2831, 1581, 1494, 1457; ^1^H NMR (400 MHz, CDCl_3_) *δ* 7.34–7.23 (20 H, m, ArC*H*), 7.03 (1H, d, *J* 9.0 Hz, ArC*H*), 6.69 (1H, d, *J* 9.0 Hz, ArC*H*), 6.68 (1H, d, *J* 12.0 Hz, HC=C*H*), 6.54 (1H, d, *J* 12.0 Hz, HC=C*H*), 6.49 (2H, s, 2 × ArC*H*), 5.20 (4H, d, *J* 7.0 Hz, OC*H*_2_), 5.12 (2H, d, *J* 7.3 Hz, OC*H*_2_), 5.11 (2H, d, *J* 8.0 Hz, OC*H*_2_), 3.83 (3H, s, OC*H*_3_), 3.65 (6H, s, 2 × OC*H*_3_); ^13^C NMR (101 MHz, CDCl_3_) *δ* 152.7 (*C*), 151.5 (*C*), 141.3 (d, *J* 6.9 Hz, *C*), 141.2 (d, *J* 7.7 Hz, *C*), 137.1 (*C*), 135.8 (d, *J* 8.5 Hz, *C*), 135.6 (d, *J* 7.5 Hz, *C*), 132.9–133.1 (m, *C*), 132.1 (*C*), 131.7 (C*H*), 128.4 (*C*H), 128.3 (*C*H), 128.3 (*C*H), 127.8 (*C*H), 127.7 (*C*H), 126.8 (*C*H), 124.4 (*C*), 124.3 (*C*H), 109.2 (*C*H), 109.1 (*C*H), 106.1 (CH), 70.0 (d, *J* 6.0 Hz, *C*H_2_), 69.7 (d, *J* 5.5 Hz, *C*H_2_), 60.7 (O*C*H_3_), 55.9 (2 × O*C*H_3_), 55.4 (sept, *J* 22.5 Hz, ^13^*C*D_3_); ^31^P NMR (162 MHz, CDCl_3_) *δ* -5.44 (d, *J* 2.5 Hz), -5.55 (d, *J* 2.5 Hz); *m*/*z* (TOF ES^+^) 857.2749 (100%, MH^+^. C_45_^13^CH_44_D_3_O_12_P_2_ requires 857.2765.

**3-[^13^CD_3_]-Methoxy-6-[(1*Z*)-2-(3,4,5-trimethoxyphenyl)ethenyl]-1,2-benzenediol-*bis*(dihydrogen phosphate), tetrasodium salt CA1-P 9**

Benzyl phosphate ester **8** (0.250 g, 0.292 mmol) was dissolved in acetonitrile (3 mL) at 0 °C. Sodium iodide (0.187 g, 0.292 mmol) and trimethylsilyl chloride (0.16 mL, 1.25 mmol) was added dropwise and the reaction left to stir for 30 min. The reaction was quenched by addition of 1% aqueous sodium thiosulfate solution (2 mL), and the acetonitrile removed under vacuum. The residue was suspended in a water / DCM mixture (5 mL, 1:1) and extracted with water (4 × 1 mL). The combined aqueous layers were evaporated, using a toluene azeotrope, re-dissolved in dry methanol (5 mL), and sodium methoxide added (0.42 mL of a 3M solution in methanol). After stirring for 6 hours, the suspension was filtered and the filtrate evaporated to give the crude material that was re-precipitated from water and ethanol to give the product **9** (0.155 g, 90%). ^1^H NMR (400 MHz, D_2_O) *δ* 7.06 (1H, d, *J* 12.2 Hz, *H*C=CH), 6.94 (1H, d, *J* 8.8 Hz, ArC*H*), 6.83 (2H, s, 2 × ArC*H*), 6.54 (1H, d, *J* 8.8 Hz, ArC*H*), 6.53 (1H, d, *J* 12.2 Hz, *H*C=CH), 3.78 (3H, s, 2 × OC*H*_3_), 3.75 (6H, s, OC*H*_3_); ^31^P NMR (162 MHz, D_2_O) *δ* 1.64, 1.00.

1. Design, synthesis and antiproliferative activity of the new conjugates of E7010 and resveratrol as tubulin polymerization inhibitors, Ahmed Kamal, Md. Ashraf, Shaik Thokhir Basha, S. M. Ali Hussaini, Shamshair Singh, M. V. P. S. Vishnuvardhan, Boppana Kiran and Balasubramanian Sridhar, *Organic and Biomolecular Chemistry*, **2016**, *14*, 1382-1394 [↑](#footnote-ref-1)
